# Anesthetic efficacy of Oraqix® versus Hurricaine® and placebo for pain control during non-surgical periodontal treatment

**DOI:** 10.4317/medoral.19202

**Published:** 2013-12-07

**Authors:** Gemma Mayor-Subirana, José Yagüe-García, Eduard Valmaseda-Castellón, Josep Arnabat-Domínguez, Leonardo Berini-Aytés, Cosme Gay-Escoda

**Affiliations:** 1 DDS. Fellow of the Master’s Degree Program in Oral Surgery and Implantology, School of Dentistry, University of Barcelona, Barcelona, Spain; 2 DDS. Master’s Degree Program in Oral Surgery and Implantology, School of Dentistry, University of Barcelona, Barcelona, Spain; 3 DDS, PhD. Professor of Oral and Maxillofacial Surgery, Director of Postgraduate Degree in Oral Surgery and Implantology, School of Dentistry, University of Barcelona, Barcelona, Spain. Researcher of the IDIBELL Institute; 4 MD, DDS. Associate Professor of Oral and Maxillofacial Surgery, School of Dentistry, University of Barcelona, Barcelona, Spain. Researcher of the IDIBELL Institute; 5 DDS, MD, PhD. Emeritus Professor of Oral and Maxillofacial Surgery, Professor of the Master’s Degree Program in Oral Surgery and Implantology, School of Dentistry, University of Barcelona, Barcelona, Spain. Researcher of the IDIBELL Institute; 6 DDS, MD, PhD. Chairman and Professor of Oral and Maxillofacial Surgery. Director of Master’s Degree Program in Oral Surgery and Implantology. School of Dentistry, University of Barcelona. Coordinator/Researcher of the IDIBELL Institute. Chief Oral and Maxillofacial Surgeon of the Teknon Medical Center, Barcelona, Spain

## Abstract

Objectives: To evaluate the efficacy of Oraqix® during scaling and root planing (SRP) in comparison with 20% benzocaine and placebo. Study Design: 15 patients requiring 4 sessions of SRP were enrolled. For each patient, Oraqix®, Hurricaine®, vaseline or no anesthetic product were randomly assigned each to a quadrant. Treatment pain was evaluated on a 100 mm Visual Analog Scale (VAS) and on a Verbal Rating Scale (VRS). The amount of product administered, the need to re-anesthetise, patient and operator satisfaction and the onset of side-effects were also recorded. Results: Oraqix® was significantly better than nothing, with a reduction of VAS score to 13.3 units, but without significant differences with Vaseline or Hurricaine®. Oraqix® was better in VRS reduction than not using any anesthetic (p=0.001) or using vaseline (p=0.024), but similar to Hurricaine® (p=0.232). Conclusions: Oraqix® effectively controls pain in SRP procedures, with few side-effects and a good acceptance on the part of patients and clinicians.

** Key words:**Controlled clinical trial, topical anesthetic, scaling and root planing.

## Introduction

It is estimated that about 52% of North American adult population is at risk of developing periodontal disease (1). Japan and Germany show similar prevalences. Treatment of this disease often includes Scaling and Root Planing (SRP), which is experienced by a significant number of patients as painful and uncomfortable. (2-4). Thus, local anesthesia is often required (40%) (5). The main anesthetic techniques used are nerve block/infiltration anesthesia either alone or in combination with topical anesthesia (6-9).

Nevertheless, more than 25% of adults are afraid of injections (10). Furthermore, the prolonged duration of the injected local anesthetic action and the sensation of tingling or numbness (mainly in lips and tongue) redu-ces the tolerance of SRP carried out under conventional local anesthetics (6,7,10).

Occasionally, topical anesthetics are used in order to reduce pain and discomfort during surgical procedures (6,9). The topical anesthetics most commonly used are 20% benzocaine dental gel and 5% lidocaine spray (11). These methods have the drawback of incomplete adhesion to the areas being treated. Thus, the topical anesthetic is washed away by saliva and part of its effect is lost. Other disadvantages of topical anesthetics are the lack of efficacy due to the insufficient ability to flow towards the deepest portion of the periodontal pocket, as well as the short duration of anesthesia, unfavourable taste and difficult application inside the periodontal pocket (6,9,12).

For all the above mentioned reasons, an effective topical anesthetic ideally should be easy to apply, with painless administration and effective for any patient who is reluctant to receive injections (7,13).

Oraqix® (Dentsply, Konstanz, Germany) is a topical anesthetic agent used in periodontal treatments for pain control. It consists of an eutectic mixture of 5% lidocaine and prilocaine (each gram contains 25-mg lidocaine and 25-mg prilocaine) (6,7,14). To the mixture of anesthetics, which already existed in the pharmaceutical market with the brand name EMLA® (15,16) (Eutectic Mixture of Local Anesthetics, AstraZeneca, Sodertalje, Sweden) thermosetting agents have been added, which make the consistency of the mixture vary from liquid to gel when temperature rises. Therefore, the anesthetic material is liquid at room temperature and turns into a gel at body temperature. Thus, by applying the anesthetic solution on the oral mucosa (with no perforation) and more specifically on the gingival pockets, Oraqix® consistency varies once administered, enabling it to remain in place (6-8,17) Oraqix® is supplied in dental cartridges that contain 1.7 g gel.; the anesthetic is applied on the gingival margin around the teeth using a 23-G blunt-tipped applicator included in the package, which is similar to intraligament local anesthetic injections (11).

Different studies carried out with Oraqix® Donaldson et al. (7), Jeffcoat et al. (13, Manner et al. 18) have evaluated its efficacy for intraoperative pain control during SRPs. However no study has so far compared the efficacy of Oraqix® with 20% benzocaine used in periodontal treatments.

Therefore, the objective of this split-mouth, double-blind, randomised controlled clinical trial was to determine the efficacy of Oraqix® during the SRP procedure and to compare this anesthetic material with 20% benzocaine and placebo. The secondary aims were to assess the amount of local anesthetic administered and product reapplication, to evaluate post-treatment pain using a Visual Analog Scale (VAS) and a Verbal Rating Scale (VRS), to assess patient and operator satisfaction and finally, to determine possible adverse affects.

## Material and Methods

A randomised double-blind clinical trial controlled with a placebo was conducted between from January 2008 to January 2009. The protocol of the study was approved by the CREC (Clinical Research Ethics Committee) of the School of Dentistry of the University of Barcelona, Spain. All included patients needed periodontal treatment in 4 quadrants. Each patient had its 4 quadrants randomly assigned to one of the following 4 options for anesthetic method: topical anesthetic with Oraqix® (Dentsply, Konstanz, Germany), topical anesthetic with Hurricaine® (Clarben, Madrid, Spain), Vaseline cream (Vaselina Cusí®, Sanofi-Synthelabo, Barcelona, Spain) and no anesthetic method. Thus, all patients received the 4 treatment modalities. The sample size was calculated using the statistics program G*Power 3.0 (19) and repeated-measures ANOVA design was also used. A VAS variance as reported for anesthetics was 3 and for inter-group variance was 14. Errors were set at α=0.05 and β=0.2. A total of 12 patients met the inclusion criteria and total sample size was determined to be 15 patients in total, in order to compensate possible dropouts.

Fifteen patients visiting the Pathology and Surgery Periodontal Unit of the Master’s Degree Program in Oral Surgery and Implantology (School of Dentistry, University of Barcelona, Barcelona, Spain) were included in the study. All participants met the inclusion criteria ( Table 1) and written informed consent was obtained in order to participate in the clinical trial.

**Table 1:**
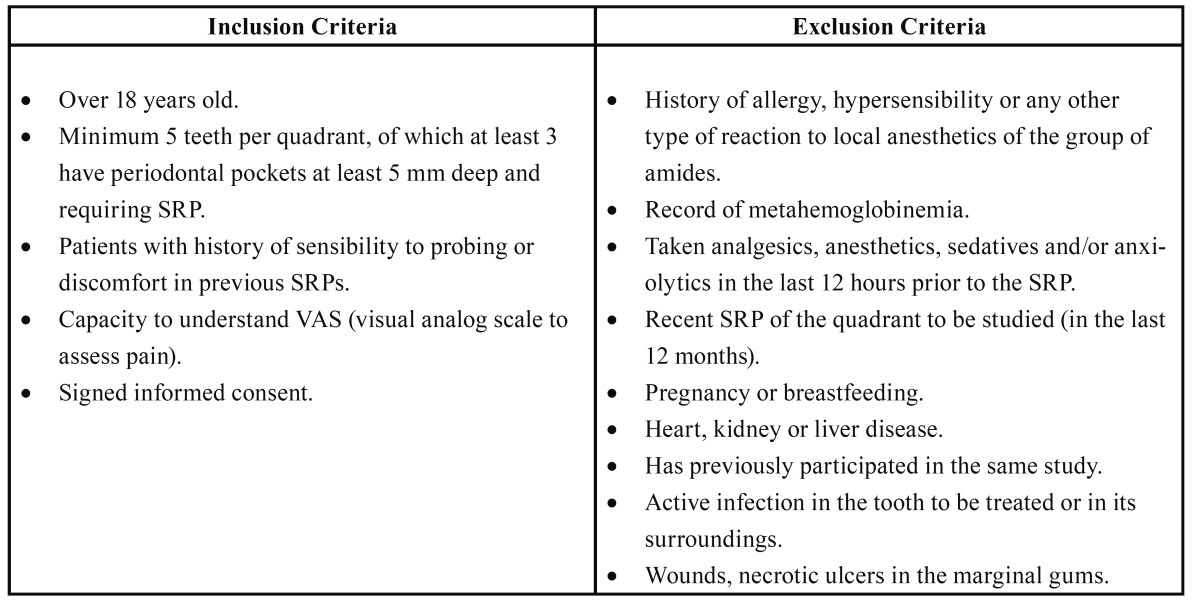
Inclusion and exclusion criteria of study participants.

All SRPs were carried out by 3 third-year fellows of the Master’s Degree Program in Oral Surgery and Implantology of University of Barcelona during four sessions, separated by 1 week. All patients were seen by the same operator to eliminate interexaminer variability.

Each patient was selected at random by means of a draw in each of the quadrants in order to identify the type of treatment to be used ( Oraqix®, Hurricaine®, Vaselina Cusí®, or no anesthetic product). The randomised treatment sequence was assigned to each patient before selection. The selected sequence was reported to the clinician that was in charge of each anesthetic technique prior to application.

**Figure 1:**
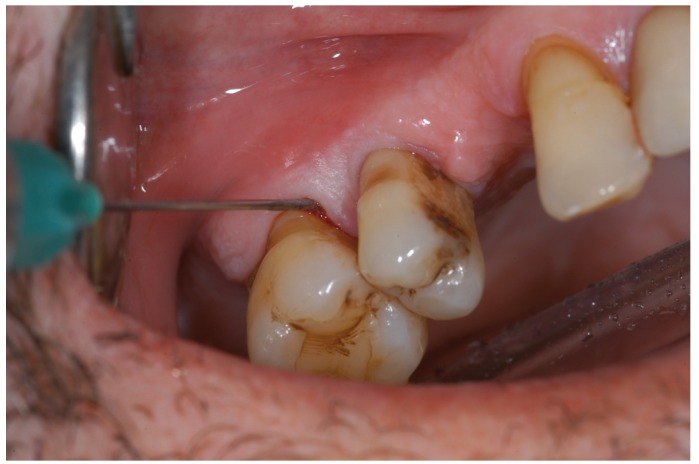
Application of Oraqix® inside the periodontal pocket.

A different operator to the one who perform the SRP, anesthetised the quadrant with the specific product assigned to it after probing each tooth of the chosen quadrant at 6 points and recording whether there was any bleeding or wound suppuration. Thus, the teeth were isolated with cotton rolls or lip separators and the selected product was placed inside the periodontal pockets from distal to mesial side. Oraqix® was introduced inside the periodontal pocket using an applicator designed for such purpose (Fig. 1). In contrast, Hurricaine® (20% benzocaine dental gel) was applied with a cotton swab and subsequently introduced into the periodontal po-cket using a probe. The same procedure was chosen for vaseline, and finally, in those quadrants where no anesthetic product was selected, the periodontal probe was only passed through the gingival margins with the aim of masking the treatment.

Following a waiting period of 30 to 45 seconds, a second operator started the instrumentation using universal and specific curettes from distal side to the halfway line. When reanesthesia was needed, the first operator placed more product on the painful area without the presence of a second operator so as not to loose the masking effect. After 30 seconds, the second operator entered the operating theatre and continued with the SRP procedure. In those areas that were not anesthetised, the same operator who had performed the SRP infiltrated the operating side with 4% Articaine with 1:100.000 Epinephrine (Inhibsa, Barcelona, Spain) as many times as needed. The amount of Oraqix® used was recorded. Patients were prescribed analgesic medication usually oral ibuprofen 600 mg, 1 granulated sachet every 8 hours (Espidifen®, Zambon Group, Vicenza, Italy).

During immediate follow-up period, within 5 minutes after treatment, the patient had to answer the following question: How much pain did you feel during the SRP procedure? The answer was marked with a vertical line on a horizontal line of 100 mm (VAS); in the far left the painless situation was found whereas the far right of the line represented the highest degree of pain. The SRP pain was also assessed using a five-point VRS (No pain, slight pain, medium pain, severe pain or very severe pain) that refers to the general degree of patient’s unpleasantness during the treatment. The taste of the anesthetic used was also recorded (Not unpleasant, unpleasant, and very unpleasant). The ease of product application by the operator was also evaluated (very easy, easy, medium or difficult) as well as the possible alteration in the operator’s sense of touch when using the curettes (none, some, quite or quite a lot).

All patients were given a questionnaire in order to identify the following adverse effects: stinging pain, irritation, soreness, swelling, loss of feeling, alteration of taste, nausea, fatigue, cephalea at 24 and 48 hours. The questionnaire was collected after 7 days during follow up visit or before the next SRP procedure.

The SPSS Inc., Chicago, IL. USA, version 15.0 statistical package was used throughout the statistical analysis (license of the University of Barcelona). A repeated-measures ANOVA design was also used for the VAS scores, the VRS scores, and the depth of the probe, in which intrasubject variability was compared across the anesthetic technique used and gender was considered a between-subjects variable. The analysis of categorical variables was carried out by χ2 tests. Analysis was intention-to-treat.

## Results

**Figure 2:**
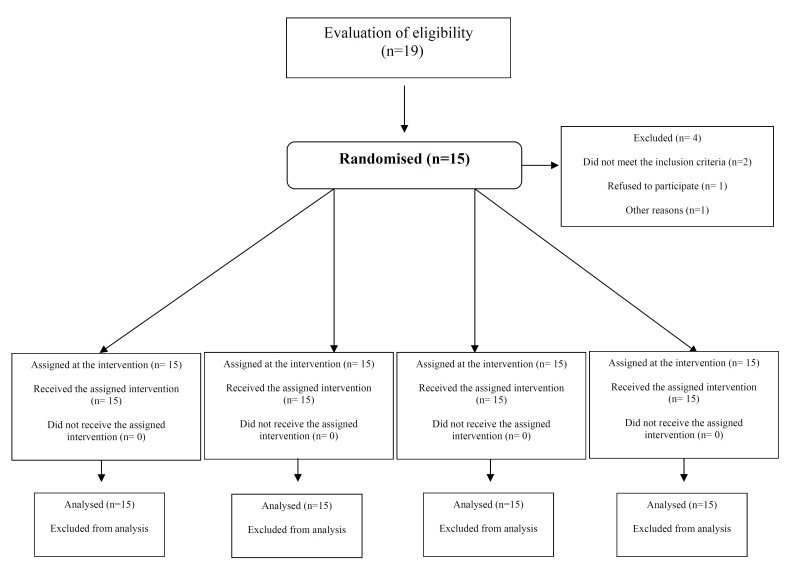
Flow chart of subjects throughout the stages of the study. There were no follow-up dropouts.

A total of 10 males and 5 females, aged 29-71, were included in the study. The mean age of the participants was 51 years. Figure 2 describes the progress of participants through the different stages of the present study. All quadrants could be treated with the initially assigned substance. No statistical significant association for the VAS scores, the VRS scores and the postoperative complications reported at 24 and 48 hours with age and gender was observed.

-Probe depth.

No statistical significant differences were observed in terms of probe depth between the 4 quadrants for each patient, nor even between different participants.  Table 2 shows probe depth measurements per quadrant for the 15 patients included in the study.

**Table 2:**
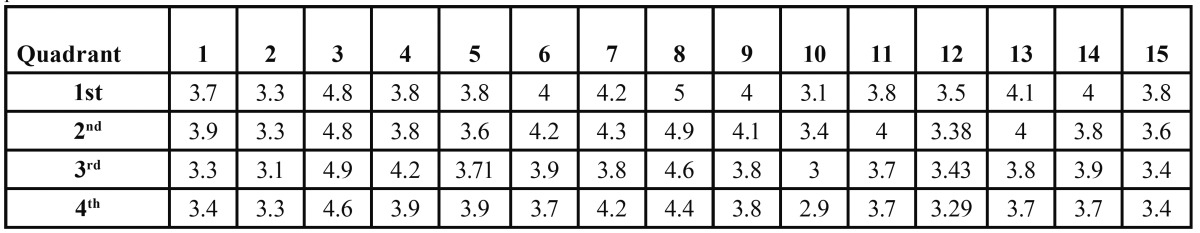
Mean probing depth measures in mm of the quadrants for the 15 patients included in the study. Each column represents a patient.

-VAS.

**Figure 3:**
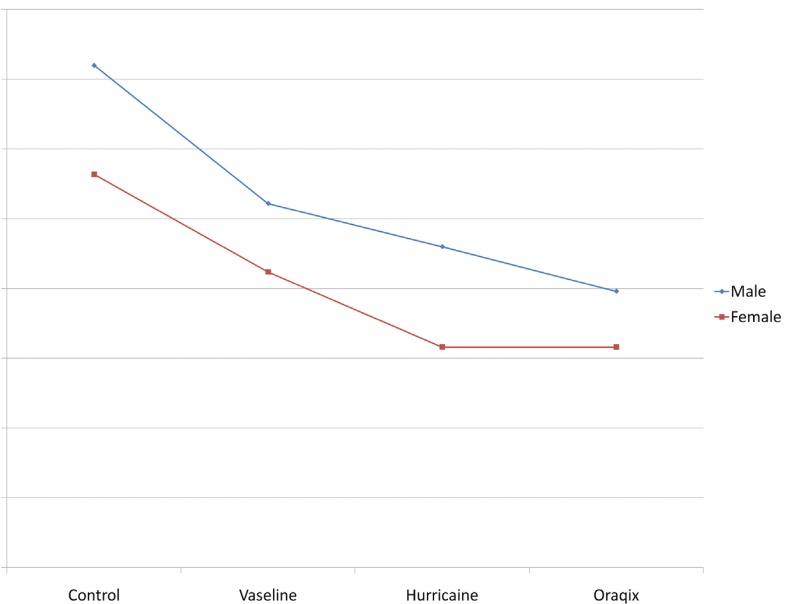
VAS scores according to type of anesthetic administered and patient’s sex.

With respect to the VAS scores, differences between groups were not significant, although they came very close to the statistical significance value (F=0.513; df= 3; p=0.053). Oraqix® proved to be significantly better than not having anything, with a reduction of 13.3 units of VAS (IC95 %: -25,6 a -3.0). Similar results were though found with Hurricaine®. There were no significant gender differences (F=0.542, df=1, p=0.475) nor interaction between gender and anesthetic type (F=0.151, df=3, p=0.927) as shown in figure 3.

-VRS.

**Figure 4:**
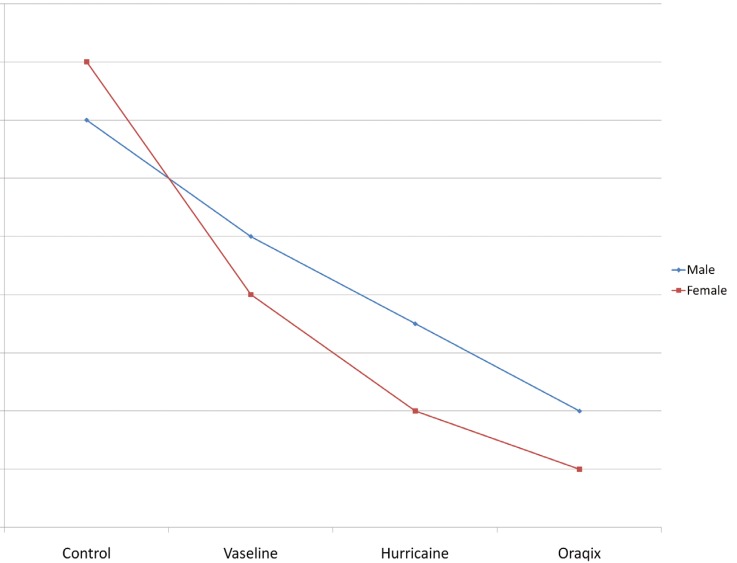
VRS scores, general degree of patient discomfort during the treatment (0= no pain, 1= slight pain, 2= medium pain, 3= severe pain, 4= very severe pain) according to topical anesthetic administered and sex.

In the VRS analysis, some statistically significant differences between groups (F=10.216, df=3; p= 4.26•10-5) were reported. Oraqix® proved better than not using any anesthetic method (p=0.001), with a reduction of 1.2 points (IC 95 %: -1.8 a -0.6) and better than applying vaseline (p=0.024), with a reduction of 0.6 points (IC 95 %: -1,1 a -0,1), but it was similar to applying Hurricaine® (p=0.232; IC 95 %: -0.6 to 0.2). There were no differences in gender (F=0,117, df=1, p=0,738) nor interaction between gender and anesthetic type (F=0.151, df=3, p=0.927). Figure 4 summarizes these results.

-Adverse effects:

Six patients treated with Oraqix® presented irritation or stinging pain at 24 hours following treatment, but it disappeared completely at 48 hour, except for one case. Four cases where no aesthetic material was administered, 4 of those treated with Hurricaine® and 3 of those who were applied Vaseline reported irritation at 24 hours. In one of the cases treated with Vaseline the irritation persisted for more than 48 hours. Only one patient reported alteration of sense of taste after the four treatment sessions, which disappeared at 48 hours.

-Amount of product administered and need to re-anesthetise:

The average dosage of Oraqix® used was ¾ to 1 cartridge per quadrant.

3 patients needed Oraqix® reapplication; after this second administration, an infiltration anesthesia was needed for just one case. In the case of Hurricaine®, reapplication was necessary in 4 cases and in 2 of them it was also necessary to administer infiltration anesthesia.  Table 3 shows the results of this section.

**Table 3:**
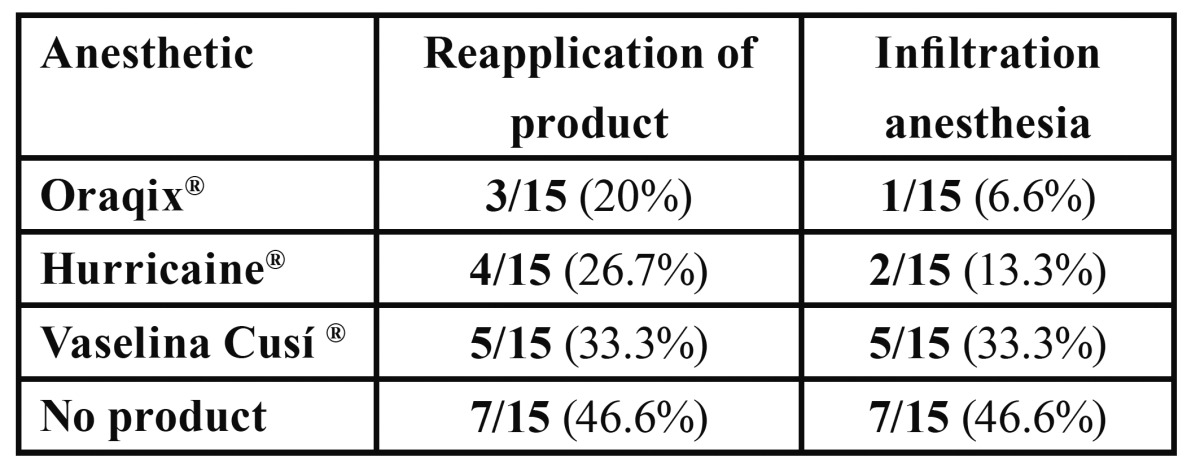
Patients who needed reapplication of anesthetic and injected anesthesia.

-Taste:

Five out of 15 patients reported an unpleasant taste when being anesthetised with Hurricaine® in contrast with 2 out of 15, who noticed an unpleasant taste after the topical anesthetic with Oraqix®. For both topical anesthetics, there was only one case that referred a very unpleasant taste. When no anesthesia was applied or just vaseline was used, all patients claimed that the taste was not unpleasant.

All patients considered that the taste would not influence their choice in future applications of these topical anesthetics during SRP maintenance. The patient who reported a very unpleasant taste with Hurricaine®, said that he would be “somewhat influenced” on being treated with this product again.

Ease of application and alteration in the operator’s sense of touch when using the curette:

 Table 4 and Table 5 show the results obtained regarding the ease of application and the alteration of the sense of touch when using the curettes.

**Table 4:**
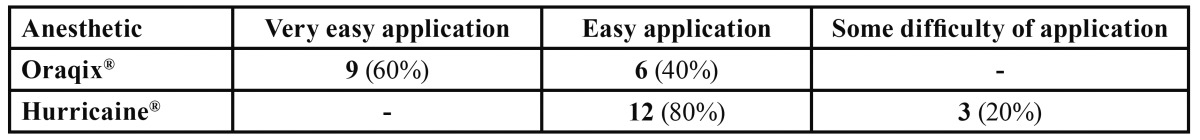
Ease of product application perceived by the clinician.

**Table 5:**
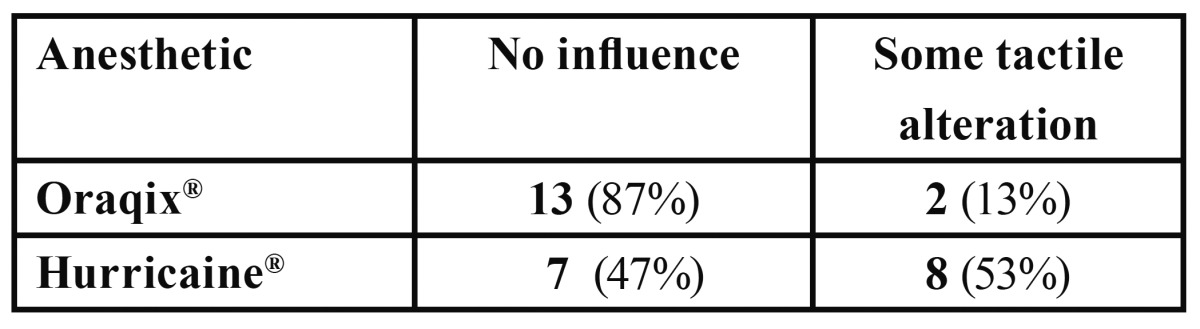
Alteration of curette touch perceived by the clinician during SRP.

## Discussion

In this study, the use of Oraqix® was associated with less intraoperative pain compared with the placebo. However no differences between the use of this anesthetic method and a 20% benzocaine dental gel were reported.

Previous studies published after the market introduction of Oraqix® have proved the efficacy of the mixture of 5% lidocaine and prilocaine (EMLA®). This topical anesthetic has been mainly used to achieve pain control during sharp debridement of chronic leg ulcers of arterial, venous or arteriovenous aetiology in Dermatology as well as for soft tissues anesthesia in children (20-22). However, the effective duration of the topical anesthetic finished at 60 minutes (23).

In Oral Surgery, EMLA® improves transepithelial transport compared with conventional topical anesthetics (24). McMillan et al. evaluated he efficacy of this anesthetic into the oral cavity (25). These authors warned the viscosity of this topical anesthesia made necessary the use of a matrix in the oral mucosa for wound therapy. Furthermore, EMLA® cream can be difficult to deliver to the oral mucosa, and 20% benzocaine gel has shown greater efficacy (26). Additionally, Magnusson et al. (27) observed that EMLA® was a more effective topical anaesthetic than 5% lidocaine gel, although its duration of action is relatively short for operations on the gingiva (around 20 minutes). In Odontopediatrics EMLA® has been used to perform minor surgeries, such as primary tooth extractions (28).

In addition, a greater efficacy of EMLA® compared with 20% benzocaine has been found in oral surgery for children. The application of EMLA® in children reduced the pain of palatal injections and placement of rubber dam clamps (15,16). Abu AlMelh et al. (12,29) concluded that topical agents based on a combination of 2.5% lidocaine and 2.5% prilocaine have shown promising results for anesthetic infiltration, as 20% benzocaine is not potent enough to eliminate pain from a needle prick.

The first studies referring to Oraqix® in scientific li-terature appeared in 2001. Friskopp et al. (6) conducted a study to determine anesthetic onset and duration of Oraqix® for SRP. Thirty patients were randomised to 30 seconds of treatment with Oraqix®. The gel was applied to periodontal pockets. Oraqix® provided anesthesia after an application time of 30 seconds, with a mean duration of action of about 17 to 20 min. For this reason, in our study the waiting period was 30-45 seconds after anesthetic application.

Friskopp and Huledal (14) described the plasma profiles of lidocaine and prilocaine following a single dose of Oraqix® to patients undergoing SRP. The total dose applied per patient was 0.9 to 3.5 g. Peak plasma concentrations of lidocaine (99–266 ng/ml) and prilocaine (46–118 ng/ml) occurred 20–40 min after the start of application.

These levels were low compared to those reported to cause initial signs of toxicity (5000–l 6000 ng/ml). Side-effects were few and mild local effects of short duration. The authors concluded that there is a large safety margin with respect to systemic effects following the application of 0.9 g to 3.5 g Oraqix® (maximum 2 cartridges) (14).Besides, the use of an increased dose of 8.5 g Oraqix (5 cartridges approximately) was well tolerated and displayed a wide safety margin with respect to plasma levels normally associated with systemic toxicity (17).

Three other multicenter studies analysed, together with our study, the efficacy of Oraqix® versus placebo as a topical anesthetic used in SRPs ( Table 6).

**Table 6:**
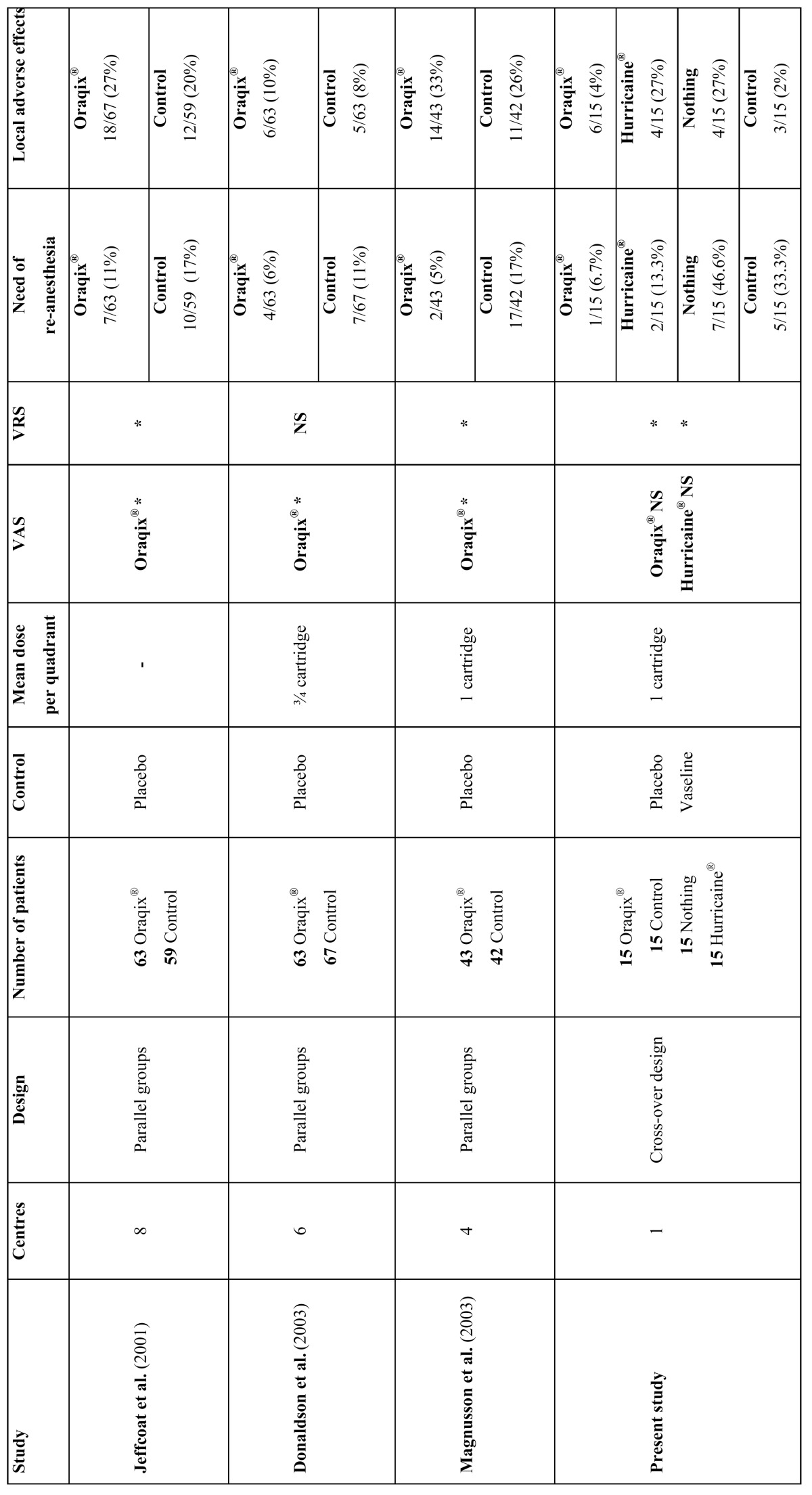
Double-blind randomized controlled trials that compare Oraqix® with placebo. ( * = statistically significant differences. NS = not significant).

In conclusion, these three multicenter studies, showed that the application of Oraqix® in periodontal pockets is well tolerated by patients and it is more effective than the placebo in reducing pain during SRP. The anesthetic gel reduced the pain by at least 50%. The median dose of active gel administered was 1 cartridge per quadrant (range from three quarters of a cartridge to 2 cartridges) (7,13,18).

The innovation of the present study lies in the fact that Oraqix® was compared to another topical anesthetic and vaseline administration. In addition, the “split-mouth” design (with intrasubject measures) reduced possible variability. Furthermore, as this was not a multicenter study, the variability caused by different treatment protocols and clinicians involved is reduced. Therefore, the sample size is considerably decreased. A problem connected with other trials is that concealment of treatment can be difficult, as placebo causes no numbness.

In a multicenter study, Van Abu Al-Melh et al. (30) assessed patients’ preference for Oraqix® versus injection anesthesia using 2% lidocaine. A total of 157 patients reporting fear to injections and requiring SRP procedure were included in the study. Two visits were randomly scheduled: at the first visit, the quadrant was anesthetised with Oraqix® whereas 2% lidocaine was applied prior to the periodontal treatment. One hundred ten out of the 157 patients (70%) reported a preference for Oraqix® due to less numbness following the procedure. Thirty-five patients (22%) preferred the injection of 2% lidocaine as they indicated less pain and other discomforts during SRP, and 8 patients (12%) did not express a preference. Ninety-six percent of patients reported that anesthesia efficacy was better with injective anesthesia compared to 80% for the gel. The researcher’s opinion on the efficacy of both anesthetic technique was similar: 100% with lidocaine versus 76% with Oraqix®. Finally, of these patients 45% would be willing to return and use the same gel. In this study, with the limitation of a smaller sample size, a greater efficacy of Oraqix® was observed as 80% of the patients and professionals expressed satisfaction with this satisfactory anesthesia with the gel. Of the 3 unsatisfied patients, with just the reapplication of Oraqix® the pain could be eased for 2 of them. In the other patient the infiltration technique had to be resorted to. Specifically, this patient required administration of local anesthetic in all 4 sessions of SRP, which suggests that topical anesthesia was not appropriate for this patient. We have only found one preliminary study conducted by Faul et al. (31) concerning the use of benzocaine as topical anesthetic during SRP procedure. The purpose of this randomised, split-mouth study conducted in 21 patients was to compare the analgesic efficacy between an intra-pocket topical 20% benzocaine gel with a blunt-tip applicator (Ultracare®, Ultradent Products Inc., South Jordan, Utah, U.S.A.) with conventional injection. Greater intra-operatorive pain was associated with the use of the gel in comparison to the conventional anesthetic, but 52% preferred topical over injected anaesthetic. In our study, 2 patients treated with Hurricaine® needed to receive anesthesia infiltration, however VAS and VRS scores obtained were similar to those obtained with Oraqix®.

In the aforementioned studies (7,13,18,30) and this preset study, the possible adverse effects arising in the different groups of patients were also assessed: those treated with Oraqix®, with placebo and with lidocaine. In all cases the number of patients presenting transitory adverse effects was similar.

In conclusion, Oraqix® is an effective gel which reduces the incidence of intraoperative pain during the SRP procedures, although there were no significant statistical differences compared with Hurricaine®. Patients treated with Oraqix® did not require another type of anesthetic. Slight unpleasant taste was reported by patients and ease of application by all operators. In 2 out of 15 cases the clinician felt a slight decrease in tactile perception when using curettes.

Therefore, the application of Oraqix® is an effective technique for pain reduction during SRPs, reporting few side effects and a good acceptance on the part of both patients and clinics. Furthermore it is a useful anesthetic method for individuals with dental anxiety and/or who are particularly sensitive to pain.
